# Double pyramidal lobe detected at ultrasound: a case report with review of the literature

**DOI:** 10.3389/fsurg.2026.1829850

**Published:** 2026-04-21

**Authors:** Spyridon Chytiris, Marsida Teliti, Laura Croce, Lidia Pizzuto, Linda Loretta Businaro, Tshering Dorji, Mario Rotondi, Benedetto Calì

**Affiliations:** 1 Unit of Endocrinology and Metabolism, Laboratory for Endocrine Disruptors, Istituti Clinici Scientifici Maugeri IRCCS, Pavia, Italy; 2 Department of Internal Medicine and Therapeutics, University of Pavia, Italy; 3 Unit of Anatomic Pathology, Istituti Clinici Scientifici Maugeri IRCCS, Pavia, Italy; 4 Department of General and Minimally Invasive Surgery, Istituti Clinici Scientifici Maugeri IRCCS, Pavia, Italy

**Keywords:** double pyramidal lobe, surgical planning, thyroid ultrasound, thyroid variant, total thyroidectomy

## Abstract

The pyramidal lobe (PL) is a common anatomical variant of the thyroid gland, arising from the caudal end of the thyroglossal duct during embryological development. Although its prevalence is approximately 40% in the general population, the presence of a double pyramidal lobe (DPL) is extremely rare. We report a unique case of preoperative ultrasound diagnosis of DPL, subsequently confirmed intraoperatively and histologically. A 61-year-old male with a long-standing toxic multinodular goiter under methimazole treatment underwent thyroid ultrasound, which revealed a markedly enlarged gland (98 mL) with bilateral nodular disease and two distinct pyramidal lobes originating from the isthmus. Fine-needle aspiration cytology showed benign findings. Due to substernal extension and tracheal deviation, total thyroidectomy was performed. Both pyramidal lobes, preoperatively identified by ultrasound, were successfully excised. Histopathological examination confirmed benign multinodular goiter without malignancy and validated the presence of two separate PLs. To our knowledge, this is the first reported case of DPL diagnosed preoperatively by ultrasound and confirmed histologically. Preoperative recognition of PL, and particularly DPL, is clinically relevant. Residual pyramidal lobe tissue after total thyroidectomy may contribute to persistent or recurrent disease. In malignancy, it can impair thyroglobulin-based surveillance and reduce radioactive iodine efficacy by competing for uptake. Given the potential surgical implications, careful preoperative ultrasound assessment by experienced operators is essential. This case highlights the importance of thorough anatomical evaluation and close collaboration between endocrinologists and surgeons to ensure complete thyroidectomy and reduce the risk of persistent or recurrent disease.

The pyramidal lobe (PL) is a common anatomical variant of the thyroid gland, arising from the caudal end of the thyroglossal duct during embryological development. Although its prevalence is approximately 40% in the general population, the presence of a double pyramidal lobe (DPL) is extremely rare. We report a unique case of preoperative ultrasound diagnosis of DPL, subsequently confirmed intraoperatively and histologically.

A 61-year-old male with a long-standing toxic multinodular goiter under methimazole treatment underwent thyroid ultrasound, which revealed a markedly enlarged gland (98 mL) with bilateral nodular disease and two distinct pyramidal lobes originating from the isthmus. Fine-needle aspiration cytology showed benign findings. Due to substernal extension and tracheal deviation, total thyroidectomy was performed. Both pyramidal lobes, preoperatively identified by ultrasound, were successfully excised. Histopathological examination confirmed benign multinodular goiter without malignancy and validated the presence of two separate PLs.

To our knowledge, this is the first reported case of DPL diagnosed preoperatively by ultrasound and confirmed histologically. Preoperative recognition of PL, and particularly DPL, is clinically relevant. Residual pyramidal lobe tissue after total thyroidectomy may contribute to persistent or recurrent disease. In malignancy, it can impair thyroglobulin-based surveillance and reduce radioactive iodine efficacy by competing for uptake. Given the potential surgical implications, careful preoperative ultrasound assessment by experienced operators is essential.

This case highlights the importance of thorough anatomical evaluation and close collaboration between endocrinologists and surgeons to ensure complete thyroidectomy and reduce the risk of persistent or recurrent disease.

## Introduction

The thyroid gland comprises two lateral lobes, right and left, connected by a central isthmus, and in some individuals, it also includes a pyramidal lobe (PL). The PL, also known as the third lobe or Lalouette's lobe, is an anatomical projection that extends superiorly, that may originate from the right, the left lobe or the isthmus of the gland and attach to the thyroid cartilage or hyoid bone (1). The PL is more commonly found in the left lobe and it more frequently terminates at the level of the thyroid cartilage (2).

The PL originates from the caudal end of thyroglossal tract during thyroid follicular descent during fetal development. Its length and size may vary, so that the PL can be characterized as short (≤15 mm), medium (16–30 mm) or long (≥31 mm).

A recent meta-analysis reports PL prevalence of 42.82%, with a slightly greater prevalence in males (40.35%) than in females (37.43%) (3). However, detection rates may vary depending on imaging techniques, operator experience, and study design. In fact, large imaging studies using computed tomography (CT) have found similar rates, with prevalence estimates ranging from 41.3% to 44.6% (2, 4). Furthermore, surgical studies report rates between 36.9% and 59.8%, while some autoptic studies suggest even higher rates, up to 80% (5–7).

High-resolution ultrasound is the gold standard imaging for the study of the thyroid gland and an adequate examination modality to detect the PL, though its sensitivity and specificity may largely vary based on the experience level and the proficiency of the examiner (7).

The PL can be involved by diseases that affect the rest of the thyroid parenchyma such as multinodular goitre (MNG), Graves’ disease or Hashimoto thyroiditis (3). More rarely, it can also harbour malignant thyroid cancer (8).

Double pyramidal lobe (DPL) is an extremely rare finding, only isolated cases have been reported in the medical literature.

We present a rare case of pre-operative diagnosis of DPL through ultrasound, confirmed intraoperatively and histologically.

## Clinical case

A 61 years old male patient, presented in our Institute with a large toxic multinodular goiter known for approximately 5 years; the patient was undergoing treatment with methimazole at the time of evaluation. Fine-needle aspiration biopsy (FNAB) was performed on the dominant nodules, yielding a benign cytological result (Bethesda 2/TIR 2) for the nodule in the right lobe and a non-diagnostic result (Bethesda 1/TIR 1) for the left lobe nodule.

At our evaluation, the patient was in a euthyroid state while undergoing antithyroid therapy (methimazole 5 mg/die). Anti-thyroglobulin antibodies, anti-thyroperoxidase antibodies and anti TSH-receptor antibodies were negative, and serum calcitonin levels were within normal limits.

**Figure 1:**
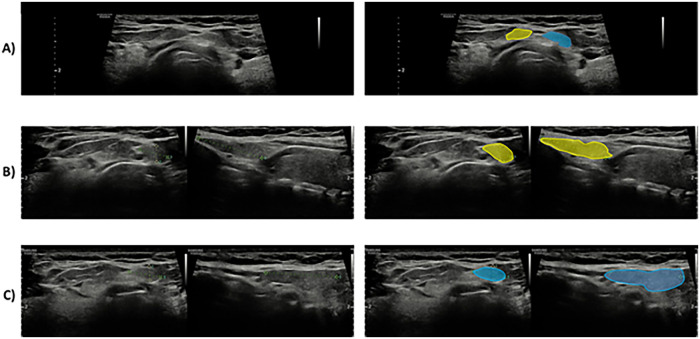
Ultrasound image of thyroid and double pyramidal lobes. In the right part of the image the two pyramidal lobes are highlighted in yellow (right) and blue (left). Panel **(A)**: Antero-posterior view. Panel **(B)** Longitudinal view of the right pyramidal lobe. Panel **(C)** Longitudinal view of the left pyramidal lobe.

Thyroid ultrasound was then performed with a 12 MHz linear transducer, which revealed an enlarged gland with an estimated volume of 98 mL. Both lobes were occupied by predominantly solid, isoechoic nodules, classified as EU-TIRADS 3. The largest nodule was located in the left lobe and measured 17 × 10 × 22 mm, showing a partially calcified peripheral rim. DPL was also observed, presenting homogeneous and normoechoic structure, and modest vascularization similar to that of the surrounding thyroid parenchyma: one spanning from the right part of the isthmus measuring 5.5 × 9.7 × 11.6 mm and the other spanning from the left part of the isthmus measuring 4.6 × 11.0 × 22.7 mm (Figure 1).

Given the presence of a large bilateral multinodular goiter with substernal extension and tracheal deviation, surgery was indicated. In this context, the choice of total thyroidectomy was due to the presence of a bilateral multinodular goiter with substernal extension and tracheal deviation, as well as for definitive treatment of hyperthyroidism.

**Figure 2:**
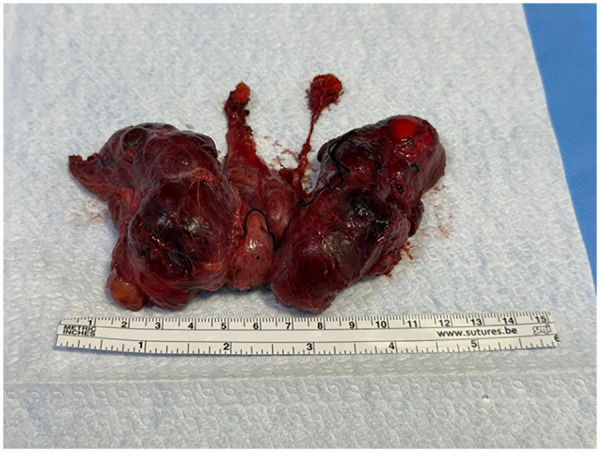
Post-operative thyroid sample.

Pre-operative ultrasound identification of the presence of this anatomical variant was considered during surgical planning. Intraoperative findings confirmed the preoperative ultrasound assessment about the presence of two distinct pyramidal lobes extending toward the hyoid bone (Figure 2). Their slender morphology would have made intraoperative identification challenging without imaging guidance. Total thyroidectomy with resection *en bloc* of both pyramidal lobes was performed by a standardized capsular dissection technique. Total surgical operative time was 110 min. No major complication occurred. A transient mild asymptomatic hypocalcaemia in the postoperative period was detected requiring supplementation with calcium carbonate and calcitriol. Drain was removed after 24 h and the patient was discharged on postoperative day two.

Histopathological examination revealed no evidence of malignancy in the two main lobes nor in the PLs. The total thyroid weight was 140 g. The length of the left PL was 1.5 cm and the right lobe was 2.5 cm. Both PLs originated from the isthmus.

Ultrasound evaluation performed nine months after surgery showed no residual thyroid tissue in typical sites of persistence, including the thyroid bed, pretracheal region, and pyramidal tract, supporting complete removal of all thyroid tissue and confirming an adequate postoperative outcome. The examination was performed by the same operator who performed the pre-surgical ultrasound exam.

## Discussion

To the best of our knowledge, this is the first reported clinical case in which a diagnosis of DPL was established preoperatively by ultrasound and subsequently confirmed by histological examination.

A DPL is a rare occurrence. Table 1 shows all published case reports of DPL detection up to now (9–16). Seven cases describe an intraoperative diagnosis of DPL without a prior ultrasound detection (9–14); only one case reported an ultrasound detection without histological DPL diagnosis, since the patient did not undergo surgery (15).

**Table 1:** case reports of double pyramidal lobe present in the literature.

References	Patients	Thyroid disease	Imaging	Surgery
Ignjatovic. 2009	54-y woman	MNG, suspicious for papillary carcinoma	Ultrasound: Multiple nodular lesions of 10–30 mm in diameter, in both lobes	TT
Hakeem et al. 2016	56-y woman	Papillary carcinoma	Ultrasound: 4 × 5 cm solid nodule in the left lobe	TT
Kaklamanos et al. 2017	45-y woman	Benign MNG	Ultrasound: Multiple solid nodules in both lobes	TT
Gürleyik. 2018	58-y woman	Toxic MNG	Ultrasound and nuclear scan: Multiple solid and hot nodules in both lobes	TT
Vithana and Rajakaruna. 2023	40-y woman	Benign MNG	Ultrasound: benign MNG with background thyroiditis without anatomical anomalies of the gland	TT
Pal et al. 2024	57-y woman	Papillary carcinoma	Ultrasound: 4 × 3 cm nodule with internal calcification and vascularity in the left lobe of the thyroid gland. The right lobe and isthmus were reported to be normal.	TT
Giordani et al. 2025	45-y woman	Benign MNG	Ultrasound: two spongiform nodules and two pyramidal lobes.	No surgery
Ağar et al, 2026 (A)	51-y woman	Papillary carcinoma	Ultrasound: 19 × 13 mm hypoechoic nodule with lobulated margins and internal microcalcifications in the left thyroid lobe.	TT
Ağar et al, 2026 (B)	55-y man	Benign MNG	Ultrasound: enlarged thyroid gland with heterogeneous parenchyma and multiple solid nodules (largest 30 mm in the right lobe and 17 mm in the left lobe)	TT
Present case	61-y-man	Toxic MNG	Ultrasound: benign MNG, a 17 × 10 × 22 mm nodule characterised by partial apical calcified rim, two pyramidal lobes	TT

MNG, multinodular goiter; TT, total thyroidectomy.

DPL is an extremely rare finding also in case series specifically evaluating the prevalence of PL with ultrasonography (17), CT (4) or in cadaveric studies (7, 18). In surgical series, a DPL was described in only 1 patient out of 445 thyroidectomized patients (8), while no case of DPL was reported by other studies (19).

The presence of the PL holds a significant clinical importance in patients undergoing surgery. Up to 10% to 30% of patients undergoing total thyroidectomy exhibit residual PL tissue (8). Incomplete excision of the PL may result in persistent or recurrent disease, even many years after total thyroidectomy, with manifestations that may be either structural (e.g., nodular regrowth) or biochemical (e.g., recurrent hyperthyroidism). Moreover, residual PL tissue may complicate postoperative surveillance based on serum thyroglobulin measurements in patients thyroidectomized for differentiated thyroid carcinoma. In this setting, incomplete resection of the PL may also impair the efficacy of radioactive iodine ablation, as residual thyroid tissue can uptake a substantial proportion of the administered isotope, thereby reducing its availability for targeting residual or metastatic disease (8, 20). When recurrence occurs, reoperation presents a higher risk of complications compared to a thorough, meticulous primary total thyroidectomy (10). Therefore, a thorough inspection of the PL's anatomy during thyroid surgery, with complete excision when necessary, is critical for ensuring optimal outcomes. However, as in the case of our patient, the PL can be so small and narrow that it can go unnoticed by surgeons, influencing the completeness of surgery. Preoperative identification is even more important in the case of a DPL, since the intraoperative detection of the second PL is usually not expected, being an extremely rare finding.

A careful ultrasound evaluation by an experienced operator, prior to surgery can improve surgical planning and be, as in our case, the critical factor for a complete thyroidectomy. Hence, the combination of ultrasound and a thorough examination of the anterior cervical region during surgery, are fundamental to ensure the removal of a DPL and therefore to reduce the likelihood of recurrence in both benign and malignant thyroid conditions.

In conclusion, the presence of a DPL, although rare, should always be preoperatively assessed with ultrasound to avoid missing it intraoperatively during total thyroidectomy. A multidisciplinary approach, with a close collaboration between endocrinologists and thyroid surgeons, is crucial to provide an effective management of this rare anatomical variant.

## Data Availability

The original contributions presented in the study are included in the article/Supplementary Material, further inquiries can be directed to the corresponding author.
